# Circulating microRNA signatures in patients with chronic Urticaria

**DOI:** 10.5339/qmj.2023.sqac.20

**Published:** 2023-11-26

**Authors:** Jayakumar Jerobin, Ramzy Mohammed Ali, Sally Khalil, Manjunath Ramanjaneya, Ilham Betahi, Abdul Badi Abou Samra, Maryam A Al-Nesf

**Affiliations:** ^1^Qatar Metabolic Institute, Academic Health System, Hamad Medical Corporation, PO Box 3050, Doha, Qatar Email: JKumar3@hamad.qa; ^2^Adult Allergy and Immunology Section, Department of Medicine, Hamad Medical Corporation, PO Box 3050, Doha, Qatar

## Abstract

**Background:** Chronic Urticaria (CU) is a complex skin disease that appears as recurrent raised itchy rash/angioedema or both for more than six weeks. The pathophysiology of CU is complex and has yet to be understood entirely. It is predominantly a mast cell-driven disease with the possible involvement of type 2 inflammation. Current evidence largely favors mast cell activation by an IgE-mediated autoallergic mechanism or an autoimmune mechanism by IgG autoantibodies to IgE/ high-affinity receptor of IgE. MicroRNAs (miRNA) are small coding RNAs regulating gene expression at the post-transcription level. This study aimed to investigate the circulating miRNA as potential biomarkers in CU patients compared to healthy controls.

**Methods:** The miRNA gene expression was done in seven patients with CU and seven healthy controls. The expression of miRNA is done using TaqMan openArray human advanced miRNA Panel. ExpressionSuite Software (Thermo Fischer Scientific, Waltham, MA, USA) is used for data analysis to quantify the miRNA expressions. P<0.05 is considered to be statistically significant.

**Results:** A significant upregulation (p<0.05) in the miR-451a, miR-9-5p, miR-150-5p, miR-296-5p, and miR-182-5p was observed in CU compared to controls. Dysregulation of miR-451a is identified as an early biomarker in allergic diseases. Functional enrichment analysis with the KEGG pathway and disease ontology databases showed that these miRNAs were associated with skin diseases and inflammation. The differentially expressed miRNAs contribute to determining the genes regulated in CU. miRNA-based therapies that target different genes in a given pathway might be a potential candidate for treating CU.

**Conclusion:** miRNA field has grown steadily over the past few years, but the role of circulating miRNAs in CU remains relatively unexplored. This study showed that the upregulated circulating miRNA might play an essential role in CU pathogenesis and inflammation. Also, our study highlights the importance of miRNAs as a future biomarker and potential therapeutic target to be investigated.
